# Supplementary material for: Brazilian Multiethnic Association Study of Genetic Variant Interactions among FOS, CASP8, MMP2 and CRISPLD2 in the Risk of Nonsyndromic Cleft Lip with or without Cleft Palate
Source: Dent J (Basel). 2022 Dec 26;11(1):7. doi: 10.3390/dj11010007 (PMC9857865; doi:10.3390/dj11010007)
Supplement: Supplementary file 1 [file dentistry-11-00007-s001.zip › dentistry-1971369-supplementary.pdf]

Table S1. Association between *FOS* rs1046117 and the risk of nonsyndromic cleft lip with or without cleft palate (NSCL±P), nonsyndromic cleft lip only (NSCLO) and nonsyndromic cleft lip and palate (NSCLP) in patients stratified by genomic ancestry: high European (A) and high African (B). *p* values were adjusted for covariates by logistic regression analysis.

A. High European Genomic Ancestry

	Control	NSCL±P	OR (95% CI)/p value	NSCLO	OR (95% CI)/p value	NSCLP	OR (95% CI)/p value
Allele							
T	79.4%	77.4%	Reference	75.7%	Reference	78.1%	Reference
C	20.6%	22.6%	1.12 (0.93-1.36)/0.23	24.3%	1.23 (0.93-1.64)/0.15	21.9%	1.08 (0.87-1.34)/0.48
Genotype							
TT	64.0%	59.2%	Reference	55.1%	Reference	62.0%	Reference
TC	30.8%	35.4%	1.20 (0.93-1.53)/0.15	41.3%	1.56 (1.09-2.22)/0.01	32.2%	1.07 (0.82-1.41)/0.60
CC	5.2%	5.4%	1.08 (0.63-1.83)/0.70	3.6%	0.81 (0.33-1.99)/0.66	5.8%	1.15 (0.66-2.01)/0.59
Dominant (TT/TC + CC)	64.0%/36.0%	59.2%/40.8%	1.18 (0.93-1.50)/0.17	55.1%/44.9%	1.45 (1.03-2.05)/0.03	62.0%/38.0%	1.08 (0.84-1.40)/0.54
Recessive (TT + TC/CC)	94.8%/5.2%	94.6%/5.4%	1.01 (0.60-1.70)/0.96	96.4%/3.6%	0.69 (0.28-1.68)/0.39	94.2%/5.8%	1.13 (0.65-1.95)/0.66

B. High African Genomic Ancestry

	Control	NSCL±P	OR (95% CI)/p value	NSCLO	OR (95% CI)/p value	NSCLP	OR (95% CI)/p value
Allele							
T	83.3%	77.7%	Reference	77.4%	Reference	77.8%	Reference
C	16.7%	22.3%	1.43 (1.01-2.03)/0.04	22.6%	1.42 (0.84-2.38)/0.18	22.2%	1.42 (0.97-2.08)/0.07
Genotype							
TT	71.5%	64.3%	Reference	60.4%	Reference	63.7%	Reference
TC	23.5%	29.0%	1.44 (0.94-2.21)/0.08	34.0%	1.81 (0.93-3.52)/0.08	28.1%	1.39 (0.84-2.30)/0.18
CC	5.0%	6.7%	1.47 (0.67-3.24)/0.34	5.7%	1.45 (0.38-5.55)/0.57	8.1%	1.86 (0.77-4.49)/0.20
Dominant (TT/TC + CC)	71.5%/28.5%	64.3%/35.7%	1.45 (0.97-2.15)/0.06	60.4%/39.7%	1.74 (0.93-3.28)/0.08	63.7%/36.2%	1.48 (0.93-2.35)/0.09
Recessive (TT + TC/CC)	95.0%/5.0%	93.3%/6.7%	1.33 (0.61-2.89)/0.47	94.4%/5.7%	1.21 (0.32-4.54)/0.78	91.8%/8.1%	1.69 (0.71-4.03)/0.23

Table S2. Association between *CASP8* rs3769825 and the risk of nonsyndromic cleft lip with or without cleft palate (NSCL±P), nonsyndromic cleft lip only (NSCLO) and nonsyndromic cleft lip and palate (NSCLP) in patients stratified by genomic ancestry: high European (A) and high African (B). *p* values were adjusted for covariates by logistic regression analysis.

A. High European Genomic Ancestry

	Control	NSCL±P	OR (95% CI)/p value	NSCLO	OR (95% CI)/p value	NSCLP	OR (95% CI)/p value
Allele							

A	52.5%	56.7%	Reference	57.9%	Reference	56.3%	Reference
G	47.5%	43.3%	0.84 (0.72-0.99)/0.04	42.1%	0.80 (0.63-1.03)/0.08	43.7%	0.86 (0.72-1.03)/0.09
Genotype							
AA	27.1%	31.1%	Reference	33.9%	Reference	31.0%	Reference
AG	50.9%	50.5%	0.85 (0.65-1.11)/0.24	47.9%	0.75 (0.51-1.10)/0.14	50.6%	0.88 (0.66-1.17)/0.37
GG	22.0%	18.4%	0.69 (0.49-0.96)/**0.02**	18.2%	0.66 (0.40-1.08)/0.10	18.4%	0.74 (0.51-1.06)/0.10
Dominant (AA/AG + GG)	27.1%/72.9%	31.1%/68.9%	0.80 (0.62-1.03)/0.08	33.9%/66.1%	0.72 (0.50-1.04)/0.08	31.0%/69.0%	0.84 (0.64-1.10)/0.19
Recessive (AA + AG/GG)	78.0%/22.0%	81.6%/18.4%	0.76 (0.57-1.02)/0.06	81.8%/18.2%	0.79 (0.51-1.22)/0.27	81.6%/18.4%	0.80 (0.59-1.10)/0.16

B. High African Genomic Ancestry

	Control	NSCL±P	OR (95% CI)/p value	NSCLO	OR (95% CI)/p value	NSCLP	OR (95% CI)/p value
Allele							
A	58.1%	57.0%	Reference	60.0%	Reference	55.8%	Reference
G	41.9%	43.0%	1.05 (0.79-1.37)/0.74	40.0%	0.93 (0.61-1.40)/0.71	44.2%	1.10 (0.81-1.48)/0.53
Genotype							
AA	32.6%	32.3%	Reference	36.7%	Reference	28.1%	Reference
AG	51.1%	51.1%	1.01 (0.68-1.52)/0.98	46.7%	0.78 (0.41-1.47)/0.43	55.5%	1.29 (0.79-2.08)/0.36
GG	16.3%	16.6%	1.11 (0.64-1.92)/0.66	16.7%	0.84 (0.36-1.98)/0.69	16.4%	1.16 (0.60-2.21)/0.64
Dominant (AA/AG + GG)	32.6%/67.4%	32.3%/67.7%	1.03 (0.70-1.52)/0.86	36.7%/63.4%	0.79 (0.44-1.45)/0.45	28.1%/71.9%	1.25 (0.79-1.99)/0.33
Recessive (AA + AG/GG)	83.7%/16.3%	83.4%/16.6%	1.10 (0.67-1.80)/0.70	83.4%/16.7%	0.98 (0.45-2.12)/0.95	83.6%/16.4%	0.99 (0.56-1.74)/0.96

Table S3. Association between *MMP2* rs243836 and the risk of nonsyndromic cleft lip with or without cleft palate (NSCL±P), nonsyndromic cleft lip only (NSCLO) and nonsyndromic cleft lip and palate (NSCLP) in patients stratified by genomic ancestry: high European (A) and high African (B). *p* values were adjusted for covariates by logistic regression analysis.

A. High European Genomic Ancestry

	Control	NSCL±P	OR (95% CI)/p value	NSCLO	OR (95% CI)/p value	NSCLP	OR (95% CI)/p value
Allele							
G	53.5%	52.1%	Reference	53.6%	Reference	51.5%	Reference
A	46.5%	47.9%	1.06 (0.90-1.24/0.49	46.4%	0.99 (0.78-1.27)/0.97	48.5%	1.08 (0.91-1.29)/0.37
Genotype							
GG	28.2%	28.0%	Reference	29.2%	Reference	26.8%	Reference
GA	50.5%	48.7%	1.00 (0.76-1.31)/0.99	48.8%	0.93 (0.62-1.39)/0.71	49.4%	1.03 (0.77-1.39)/0.80
AA	21.3%	23.3%	1.14 (0.83-1.58)/0.39	23.0%	1.01 (0.62-1.63)/0.90	23.8%	1.17 (0.82-1.66)/0.35
Dominant (GG/GA + AA)	28.2%/71.8 %	28.0%/72.0 %	1.04 (0.81-1.35)/0.74	29.2%/71.8 %	0.95 (0.66-1.39)/0.80	26.8%/73.2 %	1.07 (0.81-1.42)/0.61
Recessive (GG + GA/AA)	78.7%/21.3 %	76.7%/23.3 %	1.14 (0.87-1.51)/0.34	78.0%/23.0 %	1.06 (0.70-1.60)/0.79	76.2%/23.8 %	1.14 (0.85-1.54)/0.37

B. High African Genomic Ancestry

.	Control	NSCL±P	OR (95% CI)/p value	NSCLO	OR (95% CI)/p value	NSCLP	OR (95% CI)/p value
Allele							
G	53.8%	53.6%	Reference	54.0%	Reference	53.4%	Reference
A	46.2%	46.4%	1.01 (0.77-1.32/0.94	46.0%	0.99 (0.66-1.48)/0.97	46.6%	1.02 (0.75-1.37)/0.91
Genotype							
GG	29.0%	28.1%	Reference	30.6%	Reference	29.3%	Reference
GA	49.8%	49.4%	0.98 (0.64-1.50)/0.87	46.8%	0.91 (0.47-1.76)/0.78	48.3%	0.95 (0.58-1.55)/0.75
AA	21.3%	22.5%	1.05 (0.63-1.76)/0.80	22.6%	1.04 (0.47-2.31)/0.92	22.4%	1.06 (0.59-1.91)/0.85
Dominant (GG/GA + AA)	29.0%/71.1 %	28.1%/71.9 %	1.00 (0.67-1.49)/0.99	30.6%/69.4 %	0.95 (0.51-1.76)/0.87	29.3%/70.7 %	0.98 (0.62-1.56)/0.94
Recessive (GG + GA/AA)	78.8%/21.3 %	77.5%/22.5 %	1.07 (0.69-1.64)/0.77	77.4%/22.6 %	1.11 (0.56-2.19)/0.77	77.6%/22.4 %	1.09 (0.66-1.81)/0.73
